# Precise Pollen Grain Detection in Bright Field Microscopy Using Deep Learning Techniques

**DOI:** 10.3390/s19163583

**Published:** 2019-08-17

**Authors:** Ramón Gallardo-Caballero, Carlos J. García-Orellana, Antonio García-Manso, Horacio M. González-Velasco, Rafael Tormo-Molina, Miguel Macías-Macías

**Affiliations:** 1 Instituto de Computación Científica Avanzada (ICCAEx) and CAPI Research Group, Universidad de Extremadura, E-06006 Badajoz, Spain; 2 Department of Plant Biology, Ecology and Earth Sciences, Universidad de Extremadura, E-06006 Badajoz, Spain

**Keywords:** convolutional neural networks, deep learning, bright-field microscopy, visible light camera sensor, automated palynology

## Abstract

The determination of daily concentrations of atmospheric pollen is important in the medical and biological fields. Obtaining pollen concentrations is a complex and time-consuming task for specialized personnel. The automatic location of pollen grains is a handicap due to the high complexity of the images to be processed, with polymorphic and clumped pollen grains, dust, or debris. The purpose of this study is to analyze the feasibility of implementing a reliable pollen grain detection system based on a convolutional neural network architecture, which will be used later as a critical part of an automated pollen concentration estimation system. We used a training set of 251 videos to train our system. As the videos record the process of focusing the samples, this system makes use of the 3D information presented by several focal planes. Besides, a separate set of 135 videos (containing 1234 pollen grains of 11 pollen types) was used to evaluate detection performance. The results are promising in detection (98.54% of recall and 99.75% of precision) and location accuracy (0.89 IoU as the average value). These results suggest that this technique can provide a reliable basis for the development of an automated pollen counting system.

## 1. Introduction

The analysis of natural microscopic images is the basis of many technological developments in fields such as medicine or biology [1,2,3]. The automatic detection of objects in images captured with an automated microscope is a challenge for many types of natural samples due to the variability of the environment. The daily monitoring of atmospheric pollen concentrations is important in the management of allergenic problems, agriculture, and climate change. Multiple aerobiological units worldwide conduct manual counts of pollen grains to provide estimates of concentration in their work areas.

The most common samplers that are typically used in aerobiological studies for visual identification of pollen are Hirst-type [4]. In this kind of sampler, the particles are collected with an adhesive tape that is exposed to a constant air flow that is taken from the environment in which the sampler is placed. A different portion of the adhesive tape is exposed every hour to study the hourly evolution of the pollen concentration. This method requires the subsequent counting of the pollen grains under optical microscopy, the most common and economic system to analyze samples, according to a protocol. This task is time consuming, so multiple automatic image processing algorithms have been proposed in the bibliography to try to accelerate the process. Some proposals address the identification of pollen grains based on datasets that contain one grain per image [5,6,7,8]. The list of published works about automatic pollen segmentation is short, and the authors use traditional methods like thresholding, shape and size filtering and texture analysis [9], color similarity transformation [10], morphological operators [8,11], multi-frequency filtering [12], and active contours [13].

From the computational point of view, we are facing a problem of detecting objects (pollen grains) in an image, and this field of science has undergone a breakthrough in recent years with the proposal of novel and efficient algorithms that, to our knowledge, have not yet been applied to the localization of pollen grains. Traditionally, image classification and object detection techniques were based on a user-defined feature extraction stage followed by a classification algorithm, generally a multi-layer perceptron neural network or an SVM. The emergence of convolutional neural networks (CNN) based on deep learning has been a radical change when facing the tasks of detection and classification of images.

A drawback of deep CNNs is that they have many adjustable parameters, and therefore, they require a large set of prototypes to be trained efficiently. It would be difficult to carry out the task of manually labeling millions of palynological images, to train a network specifically adjusted from scratch to this type of image. Even so, if the set of prototypes is limited, we can make use of the transfer-learning technique [14] to try to address the problem. In any case, training of networks based on deep learning requires high computing capabilities. This fact, that posed a great inconvenience some years ago, has been mitigated by the use of modern graphic cards (GPU) that allow the implementation of this type of algorithms in reasonable times.

In recent years, CNNs have been used successfully in both classification tasks and object detection. The first proposal that aroused interest as an object detection system based on CNN was regions with CNN (R-CNN) [15]. Later, some algorithms like Fast R-CNN [16], Faster R-CNN [17], Mask R-CNN [18], or RetinaNet [19] were developed to detect objects in images with more accuracy and were notably faster. The time required by the Faster R-CNN algorithm to process an image makes it suitable for real-time object detection applications, where the identification of a precise edge is not required. The processing speed shown on macroscopic datasets by the Faster R-CNN algorithm leads us to select it to try to solve one of the problems associated with pollen detection: the need to analyze several focal planes per sample.

Our research is novel in the following aspects compared to the referenced previous works:

- This study is the first to apply a CNN-based method to standard airborne pollen slides to perform pollen detection.
- In spite of the variable complexity of the backgrounds, the proposed system does not require any pre-processing of the input images for the detection of pollen grains.
- We use multifocal information to improve the detection performance, despite the imperfections of the adhesive substrate, which make the grains appear focused at distant focal planes. In addition, this procedure allows us to use the surface ornamentation information of the grain to locate some types of pollen.
- We used a standard overlapping criterion to determine the existence of a successful detection. This choice eliminates the bias that may be introduced by other non-standard detection criteria. With this criterion, we provide a first reference in detection, valid for the 11 pollen types studied.
- To make performance comparison experiments possible, our custom-made database, built from 20 slides of 11 pollen types, has been made public to other researchers as specified in the Supplementary Materials.

This paper is organized as follows. Previous related works are presented and reviewed in Section 2. We then provide in Section 3 our collected and processed dataset, the neural infrastructure, and implementation details. Section 4 shows numerical and graphical results. Finally, the discussion, conclusions, and future works are presented in Section 5.

## 2. Related Works

To our knowledge, the most recent publications in this field exclusively address the classification of isolated grains. Among them, we can highlight some that use deep learning techniques, either using a single image per grain [5,7] or using z-stacks for a single pollen grain [20]. The reported experiments are usually performed with self-collected databases; however, at least two public databases for grain classification exist, Duller’s Pollen Dataset [21] and POLEN23E [22]. Duller’s database contains a total of 630 grayscale images of a size of 25 × 25. POLEN23E database, which was used in [5], consisting of 805 images of 23 pollen types, providing 35 color samples for each pollen type, with at least 250 pixels for each dimension.

Despite the existence of these databases, they cannot be used in detection, as pollen grains are already segmented. We have not located a database suitable for grain detection purposes. For that reason, the works analyzed below only used self-collected databases, which makes comparisons difficult.

There are large differences in the size and characteristics of the set of samples used by each of the referenced studies. Automatic grain localization in pure samples with isolated objects yields good results, with high recall and precision, but when the sample is more realistic, with grouped or clumped pollen grains and debris, the performance indicators of the proposed systems decrease [9]. Table 1 shows some data of interest of the most recent works in which they carried out the detection of pollen grains using microscopic images. As can be seen, the studies with a smaller number of grains to locate, which also report higher accuracy rates, were those of Landsmeer et al. [10] and Nguyen et al. [13].

Landsmeer et al. used a set of 44 images of successive focal planes to train their system. Firstly, the system used a color-based transformation on the images to convert the color of each pixel to a numerical value. Next, they used circular Hough transform to infer circular objects in the image. After a color filtering and object clustering, it established the grain proposals. To evaluate the performance of the system, they used 17 image stacks, containing a total of 65 pollen grains and reporting a recall rate of 86% and an accuracy of 61%.

Nguyen et al. used a single slide scanned at 40× magnification and collected a total of 768 grains from nine pollen types. To locate the edge of each pollen grain accurately, they used active contours, estimating the initial contours by means of the circular Hough transform. In the evaluation of the detection performance, they considered success if the distance to the nearest grain manually labeled was within the diameter of the smallest pollen type. With this criterion, they reported that their method performed at 93.8% recall and 89.5% precision.

Redondo et al. [8] conducted a study on bee pollen where they tried to segment the grains of 15 pollen types automatically, using morphological operators and, later, a manual outline correction. Although the number of grains used was high, they did not provide performance metrics for their segmentation algorithm.

Ranzato et al. [12] conducted an experiment to detect biological particles in microscopic images. They developed their classifier using a dataset of microscopic particles found in urinalysis and tested it on a dataset of airborne pollen images. The pollen dataset had 1429 images containing 3686 pollen grains belonging to 27 species. Its detection algorithm was based on Lowe’s object recognition from local scale-invariant features [23], reporting a recall rate of 93.9% over the pollen dataset, at the expense of a detection accuracy of 8.6%.

Finally, Diaz-Lopez et al. [9] developed a grain detection system that generated grain proposals through five steps. The algorithm first performed a background removal. Next, it selected the pink objects by color filtering. Then, it discarded objects with low eccentricity and those that could not be pollen grains due to their size. Finally, they used a classifier that, based on characteristics of color, shape, and texture, generated the grain proposals. To measure performance, they used 12 airborne pollen samples containing 3999 grains counted by an expert. There was no express mention of the use of these samples to adjust the system classifier. As final results, they reported an 81.92% recall rate with an accuracy of 18.5%. Again, we can see a low accuracy rate when using a large dataset, being insufficient values to develop an automated palynological system, as the authors concluded.

Both the heterogeneity of the datasets and the different descriptions of the correct location concept complicate the task of making a meaningful comparison between the analyzed works, or with our own results. Moreover, a reliable reproduction of the proposed detection algorithms on our dataset is very difficult, since the configuration parameters are not always completely detailed in the most recent referenced works.

## 3. Materials and Methods

### 3.1. Database

**Figure 1:**
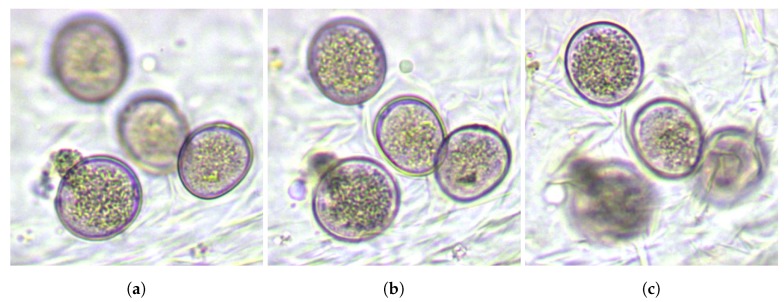
Different focal planes of the same area of a sample. It can be observed how the selected focal plane significantly influences the identification of grain-type objects: (**a**,**c**) show highly unfocused grains, while (**b**) allows the four grains to be identified.

Two main difficulties are associated with automated pollen recognition in bright-field microscopy [24]: images partially focused over several pollen grains and multiple grain orientation possibilities, both due to 2D captures of volumetric objects. A single view of a pollen slide may hide surface ornamentation of the grains, or in the worst case, the grain may not be visible, as shown in Figure 1. Hence, a multifocal analysis approach can increase the possibilities of a successful identification. Generally, pollen grain sizes vary in the range of 8–100 μm and visually appear as spheroidal objects. In addition to pollen grains, natural samples contain dust, debris, and spores, which can also have a spherical appearance and complicate the task of efficiently locating the different pollen species automatically.

**Figure 2:**
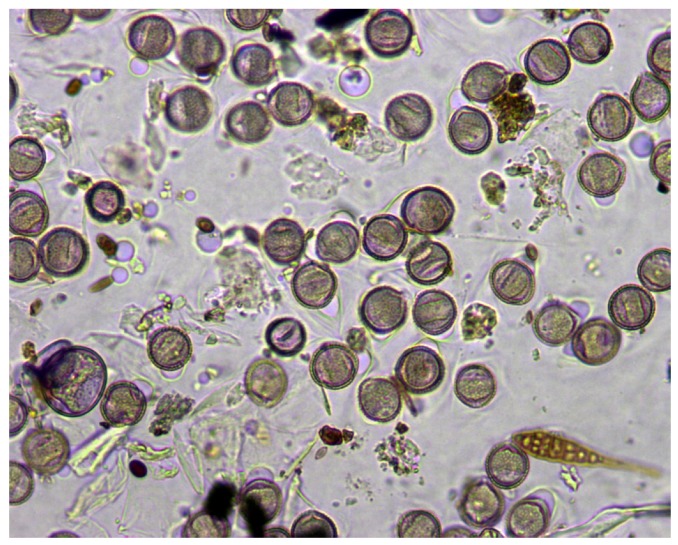
Sample input frame. Observe overlapped and blurred grains in the lower left area. The adhesive residues and tinted bubbles that appear in the background complicate the processing of the sample.

To deal with three-dimensional visual information of pollen grains, we recorded the process of focusing each sample in mjpeg video format. In this way, we can process each focal plane in the z-axis to locate pollen grains taking advantage of the information provided by the surface ornamentation of the grains, which appears when the focal plane varies along the z-axis. This work is based on a set of monospecies slides prepared in the laboratory to facilitate the tasks of labeling the objects of interest present in each sample. To reinforce the robustness of the system, some samples showed the usual imperfections in real daily airborne samples: grouped or clumped grains, bubbles, debris, and spores, as shown in Figure 2. Pollen slides were colored with fuchsia and originally captured at 1280 × 1024 RGB pixels with a 40× magnification. From each slide, we recorded from 15–40 videos (samples), depending on the concentration of grains present in the sample, obtaining a final set of 406 samples of 11 different pollen species listed in Table 2.

Table 2 also exposes the number of grains labeled on all the samples taken from the slides and how many of those grains are seen completely in the picture. Each grain was defined in a sample by a bounding box (BBox) in the focal plane (video frame) where the grain border was sharpest. This way, each sample can have bounding boxes defined in various focal planes to cover all grains contained in the view. Obviously, the samples may contain partially-visible grains located at the edges of the image, so Table 1 reflects separately how many of the grains labeled are fully visible in the views used in this work.

As we explained above, we need to process various frames for each sample to use ornamentation information, but the position and size of each grain varies depending on the focal plane. Moreover, a marked grain in a sample may not be visible in a plane where other grains do appear, as shown in Figure 1. The location algorithm that we planned to use requires as input a focal plane along with a list of regions of the image containing the effectively visible grains to be located. Therefore, to achieve an efficient training of the network, we must provide for each focal plane the precise location of the objects present in the view, eliminating those that are no longer visible or appear blurred.

Given that it is unfeasible to define the position of all the grains present in each of the available focal planes in each video, we decided to use exclusively those focal planes that provide a relevant view for the identification of the pollen grains. In this way, a training frame of interest is only created if it provides a view where grain ornamentation is visible, but hidden in a previously-considered training frame.

Our system is only designed to recognize full grains; hence, although we labeled partially-visible grains in the border of the samples, these grains were not used in the training processes. Obviously, in a functional system, a partial grain not considered in a sample will be detected while sliding the sampling window in the x and y axes with enough overlap, so the fact of ignoring these grains when training is not a problem in a real environment.

Since our work uses samples that usually contain more than one grain without further preprocessing, unlike other approaches that use segmented grains as input, we must perform a reasonable distribution of the available samples between the training and test sets. The visual complexity of the input videos was variable, so that some present a high number of grains, overlapping grains, or multiple grains only observable in distant planes. However, other videos only presented one or two grains in the view under study and only required a frame to locate the grain successfully. Therefore, we made a distribution of the samples to try to achieve an overall proportion of 60% of the grains for the training set and the remaining 40% for the test set and simultaneously matched the complexity of both sets.

The allocation procedure began by sorting the videos of each pollen type by the number of grains it contained in descending order. Next, for each pollen type, we assigned every video to one of the sets in an orderly manner, trying not to exceed the pre-established grain ratio between the two sets. In Table 3, we summarize the composition of the training and test sets in terms of the number of samples assigned to each one, total grains included in each set, and the number of frames of interest defined. The high number of frames of interest reflected in the test set was due to the fact that we used at least 21 focal planes of each video to perform the recognition of grains, as will be explained in Section 3.4.

### 3.2. Neural Framework and Training

The appearance of deep learning APIs such as TensorFlow [25], Torch [26], or Caffe [27] facilitates the rapid development of deep learning algorithms in both CPU and GPU. In this work, we used the Detectron framework [28], developed with the Caffe2 API, which is now part of PyTorch [29]. The design goal of Detectron is to be flexible in order to support the rapid implementation and evaluation of research projects. This framework includes, under a common configuration system, implementations of state-of-the-art object detection algorithms such as Faster R-CNN, RetinaNet, or Mask R-CNN. Some Detectron operators do not have CPU implementations; hence, a GPU system is required for detection. Fortunately, the cost of this type of computing systems was reduced considerably, and a simple consumer-level graphic card with a cost lower than a laptop allowed the algorithm to be executed. In this work, we used a GTX 1070 Ti GPU with 8 GB of RAM. If a compact system is required in production, the algorithm can be run on a Jetson platform [30], which additionally provides a set of GPIOs to manage the microscope’s servomotors.

The experiments, performed with Faster R-CNN on the PASCAL VOC [31] and MS COCO [32] datasets achieved high object detection accuracy at a mean rate of 5 frames per second. The speed of processing achieved and the results obtained in detection led us to consider the possibility of using the algorithm to address our problem. However, two handicaps should be studied in our implementation: the type of images and a low number of images to train. The images contained in the mentioned sets are images of daily life: person, bicycle, car, motorcycle, etc. On the other hand, in our work, we had a relatively small number of images labeled by hand, which were substantially different from the image space originally used in the Faster R-CNN algorithm.

**Figure 3:**
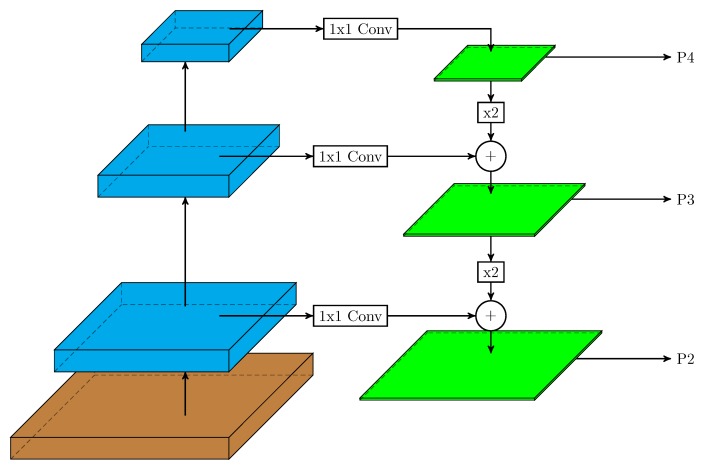
The structure of a feature pyramid network. The bottom-up pathway is a deep convolutional network. The top-down pathway combines semantically-strong features with more accurately-localized features via lateral connections.

In this work, we used a two-stage detector (Faster R-CNN with a feature pyramid network (FPN)) [33] and a one-stage detector (RetinaNet) to evaluate their performance in pollen grain detection over our database. In an FPN, the inherent multi-scale, pyramidal shape of deep convolutional networks was used to generate a feature pyramid. The general structure of this network can be seen in Figure 3. In it, the bottom-up pathway (left layers) is the convolutional network backbone that computes a hierarchy of feature maps for an input image. In the top-down pathway (right layers), the pyramid combines, at each pyramid level, low-resolution, and semantically-strong features with lower semantics, but more accurately-localized features (via lateral connections with the backbone network) to construct a rich, multi-scale feature pyramid (green layers in Figure 3). Thereby, each level of the pyramid can be used to detect objects at a different scale.

Faster R-CNN is a detection algorithm composed of three neural networks: feature network, region proposal network (RPN), and detection network. The feature network is a robust and well-tested pre-trained network such as ResNet50 [34], usually referred to as the backbone. The function of this network is to provide a good set of features from the input image. The RPN is usually a small network to generate a number of regions of interest (ROIs) that have a high probability of containing an object. Finally, the detection network (Fast R-CNN head) uses the ROIs and the feature map layer to generate a bounding box and assign it a class.

In a Faster R-CNN network with an FPN [33], the RPN is adapted by replacing the single-scale feature map obtained from the backbone with an FPN built on the backbone. In this way, both the RPN and the Fast R-CNN head use the same network backbone to allow feature sharing. Therefore, the use of an FPN will generate additional layers that use the convolutional backbone. The output of the FPN was used as the input of the RPN to generate ROIs, selecting the most proper FPN layer scale based on the size of the ROI. After ROI pooling, the output was fed into the Fast R-CNN head to finish the prediction.

Unlike two-stage networks, a one-stage network operates on a regular, densely-sampled set of possible object locations. This type of arrangement has the potential to operate faster than a two-stage network because of its simplicity. RetinaNet [19] is a one-stage unified network architecture that tries to overcome the accuracy of two-stage detectors, while maintaining a simple structure. It is composed of a backbone and two subnets and uses focal loss (FL) to concentrate training on a sparse set of hard examples, preventing the high number of easy negatives from overwhelming the detector during training. The reference RetinaNet architecture uses an FPN backbone on top of a feed-forward ResNet architecture to generate a feature pyramid. A classification subnet uses the FPN output to predict the probability of object presence at anchor positions, using FL as the loss function. Additionally, a second subnet performs regression from anchor boxes to ground-truth object boxes using a smooth L1 loss function.

**Figure 4:**
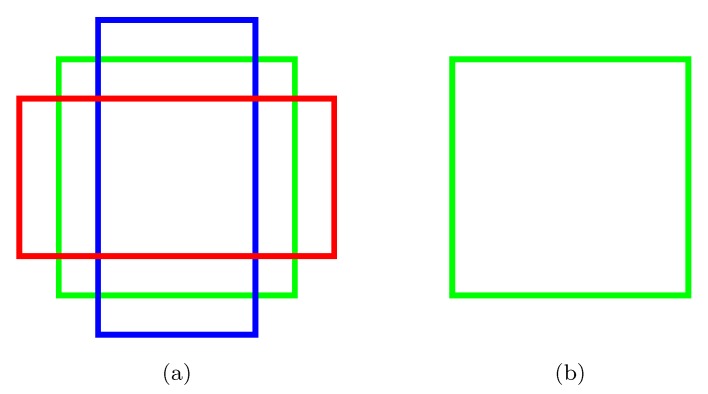
(**a**) Default Faster R-CNN anchors with aspect ratios 1:1 (green), 1:2 (blue), and 2:1 (red). (**b**) Base anchor used in our implementation with aspect ratio 1:1.

The standard approach of the Faster R-CNN algorithm uses three anchor shapes, as shown in Figure 4a. Since pollen grains are basically circular in the images, we decided to use only a square anchor shape (Figure 4b). RetinaNet also uses anchors with identical aspect ratios, so in this case, we will also apply the above criteria. Additionally, we reduced the number of categories to just perform object separation from background. The output of the network after processing an image consisted of a set of object proposals, defined by a BBox, with an associated confidence level (score).

### 3.3. Location Accuracy: Measuring the Overlap

**Figure 5:**
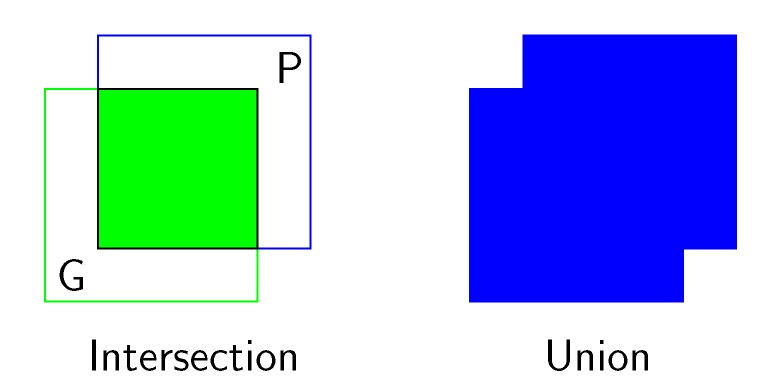
Graphical definition of intersection over the union between a ground-truth bounding box (G) containing a pollen grain and the location (P) of the grain predicted by the network.

To measure the accuracy in the proposed location of an object with respect to a given ground-truth bounding box, the intersection over the union (IoU) is usually used [35]. Intersection over the union is a simple ratio, as shown in Equation (1), where the numerator is the area of overlap between the predicted BBox (P) and the ground-truth BBox (G), and the denominator is the area encompassed by both the predicted BBox and the ground-truth BBox, as shown in Figure 5. Hence, predicted bounding boxes that heavily overlap with the ground-truth bounding boxes have higher scores than those with less overlap.

(1) $$IoU\left(P,G\right)=\frac{\left|P\cap G\right|}{\left|P\cup G\right|}$$

**Figure 6:**
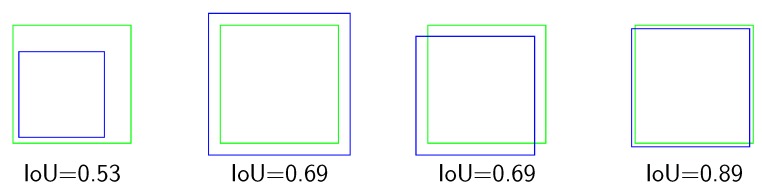
Multiple overlapping configurations and their calculated IoU ratio. In green, the ground-truth bounding box; in blue, different proposals of the bounding box.

For reference, an IoU score >0.5 is generally considered a good matched object [35]. Figure 6 shows a set of possible overlapping configurations between different proposals (blue) that could generate a detection system and a given ground-truth BBox (green). As can be seen, a value of IoU that slightly exceeds the threshold of 0.5 may not be indicative of a high-accuracy situation in terms of correctly representing the size of a pollen grain. When approaching values close to 0.9, it can be considered that the accuracy in size and location is high.

### 3.4. Merging Multifocal Information

**Figure 7:**
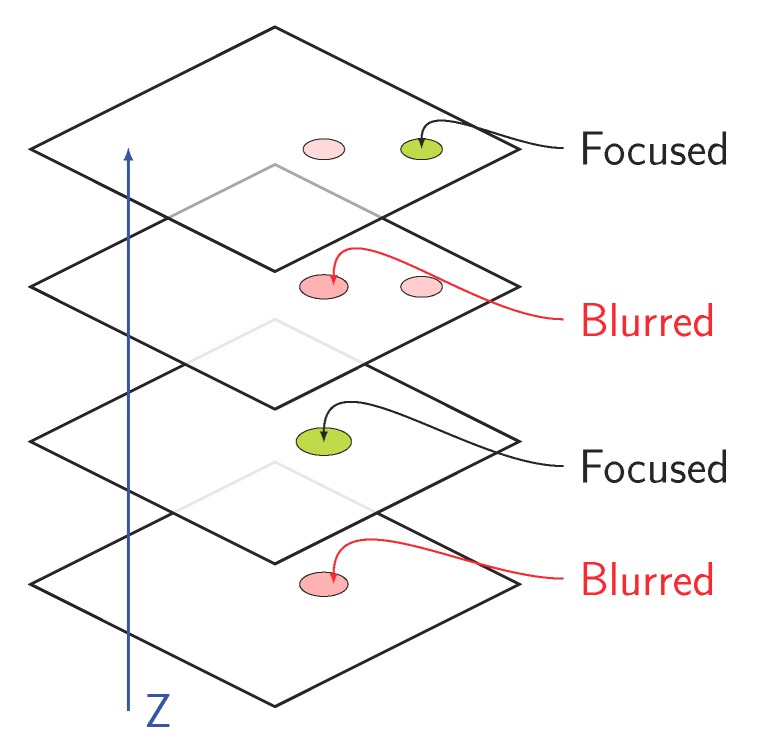
Identifiable grains in the different focal planes to be analyzed.

As we mentioned in Section 3.1, for each sample under study, we considered several video frames along the microscope’s z-axis to search thoroughly for grains. If the classifier was robust, we obtained all the visible grains in each frame presented to the network. Taking into account the grains found in all the processed frames, we can find all the grains present in a sample, regardless of their visibility or level of detail in a specific focal plane, as shown in Figure 7.

In the recognition phase, a reference frame was determined by autofocus on the video, and we processed 10 frames below this position and another 10 above it. Hence, each sample was processed in at least 21 different focal planes (video frames), and as a result, we obtained a list of bounding boxes associated with the proposals made by the network for each video frame. Next, we concatenated all the proposals in a single list *G* ordered by score, in order to apply a non-maximum suppression (NMS) algorithm. The position and the size of the grains in the visual field varied slightly when changing the focus of the microscope, thus reducing the efficiency of NMS. This effect can increase the false negative rate depending on the quality of the microscope in its z-axis travel.

**Figure 8:**
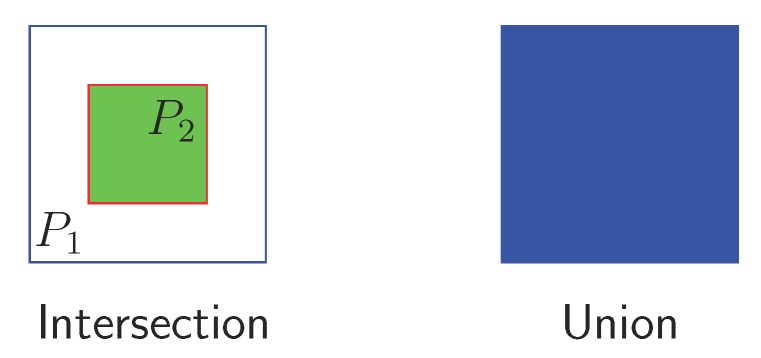
Intersection over the union when a small bounding box ($P_{2}$) is totally included within a large one ($P_{1}$). The area of the intersection corresponds to the area of $P_{2}$.

Intersection over the union is commonly used in NMS algorithms to determine the amount of overlap between the different proposals generated by a network. However, it may not detect two predictions as overlapping if one is included within another and the size difference between them is very large. In these cases, the area of the intersection corresponds to the area of the smallest proposal as shown in Figure 8, and when dividing by the area of the union, the result may be lower than the level used to suppress the proposal. As a result, both grain proposals would be evaluated to calculate the performance of the system, worsening the performance metrics. The type of images we analyzed presented these kinds of arrangement, so we made a small modification to the standard IoU definition.

Our implementation of NMS used the list of grain proposals *G*, generated by the network from all the frames considered in the sample (video), and sorted them by score in descending order. After selecting the proposal with the maximum score $M_{0}$, we eliminated any proposal that had an overlap greater than a threshold $N_{t}$ with $M_{0}$ in the set *G*. This process was repeated for remaining boxes in *G* to obtain the list of final detections *D*. However, the overlapping metric provided a total overlap value (1.0) when the area of the intersection corresponded to the area of any of the compared boxes, as shown in Equation (2).

(2) $$IoU\left(P,G\right)=\left\{\begin{array}{cc} 1.0 & \mathrm{if}\;|P\cap G|=P,\;\mathrm{or}\;|P\cap G|=G\\ \frac{\left|P\cap G\right|}{\left|P\cup G\right|} & \mathrm{otherwise} \end{array}\right.$$

### 3.5. Measuring Performance

The performance of the system was expressed by measuring recall, precision, and the IoU of the final system proposals. These parameters were calculated on all the video samples in the test set described in Section 3.1. The only preprocessing performed on the input frames was a scaling to 640 × 512. The 21 focal planes selected in each video sample were processed to simulate the sampling on the Zaxis around a reference position that was performed by the palynologist manually with the microscope.

In determining the performance of our system, we considered a true positive (TP) when a grain prediction had an IoU with the ground-truth BBox of one of the grains stored in the database of at least 0.5. A false negative (FN) was counted if none of the proposals generated by the system had the minimum overlap required in terms of IoU for a given grain. Finally, a false positive (FP) was counted whenever a proposal generated by the system did not present the minimum overlap required with any of the ground-truth bounding boxes defined in the database.

Although partial grains located at the edges of the frames were not used to adjust the classifiers, it can generate grain proposals at the edges. In these cases, the proposal was not considered a false positive (FP) if it really covered a partially-visible grain marked in the database, and was labeled as true positive at the edge ($TP_{E}$). Obviously, when moving the position of the sample in the microscope, the pollen grain would appear complete in a new sample in the X or Y direction, and then, it would be counted as TP.

The rates of IoU were calculated as the average, and for each of the species studied, with the aim of identifying possible failures associated with a given species’ visual appearance. A good performance in the adjustment of the BBox would allow providing estimates of the size of the pollen grains.

### 3.6. Network Configurations

By varying the focal plane, the grains will appear blurred in some views, and the network must correctly handle these situations. The inclusion of example grains that had a slightly blurred appearance in a given focal plane can affect the performance of the system. In order to accurately assess this impact, two different types of experiments were configured. On the one hand, experiments of Type A used all the grain prototypes defined in the training set to adjust the system, regardless of whether they provided a fuzzy or a sharp view of the pollen grain. This setting will provide networks of Type A.

On the other hand, experiments of Type B only used those examples of pollen grains that provided a sharp edge in the focal plane, discarding any grain with a blurred appearance to train. In this second case, the number of grains used in the adjustment of the system was smaller, which increased, a priori, the possibility of a reduction in yield, both in the determination of the location and in the estimation of the grain size. This setting will provide networks of Type B.

Training of both models was done in the referenced single GPU. To train the Faster R-CNN model with FPN [33] end-to-end, we used Detectron’s ResNet50 reference network as the backbone. The solver was configured to use minibatch stochastic gradient descent (SGD) with 2 images per GPU and 256 ROIs per image; hence, the total number of ROIs per training minibatch was 512. We used a weight decay of 0.0001 and momentum of 0.9. A linear warming factor of 1/3 was applied to the learning rate for the first 500 training iterations to avoid early rapid changes. The model was trained for 60,000 iterations with an initial learning rate of 0.0025, which was then divided by 10 at 20,000 and again at 40,000 iterations. Therefore, 120,000 images were seen during the training phase, which would equal around 241 epochs. Besides, we used horizontal image flipping for data augmentation. The FPN-based RPN uses pyramid levels from 2–5 and a single square aspect ratio, as previously mentioned, with a minimum anchor size of 32 pixels.

The RetinaNet model was also trained end-to-end with SGD, and the same image size and solver parameters were used. The FPN from [33] based on ResNet50 was used as the backbone network. We configured the model to consider a single squared anchor per location spanning 3 sub-octave scales on Pyramid Levels 3–7. The focal loss parameters were adjusted to the recommended values in [19] (α=0.25 and γ=2.0), and the smooth L1 loss beta for BBox regression was set to 0.11.

## 4. Results

### 4.1. Training and Detection Times

The training time for Faster R-CNN networks was just over three hours. On the other hand, the training of RetinaNet networks during the same number of iterations and on the same input dataset was over seven hours.

Once the networks were trained, we carried out the recognition of the images included in the test set. The total inference time for the 2863 images in the test set was 166.7 s for the Faster R-CNN Type A and 169.8 s for Network B of the same architecture. Hence, the processing speed was less than 59 ms per image in our GPU. In this way, the processing of the 21 frames of each sample would require less than 1.24 s in a GPU.

Performing the same analysis on the RetinaNet variants, we observed an increase in the average inference times over the test set. For the RetinaNet Type A, the average inference time was 98 ms per image, and for RetinaNet Type B, 103 ms, which would result in a minimum analysis time for each sample of just over two seconds. Table 4 shows in a compact way the training and inference times for each of the networks considered.

### 4.2. Detection Performance of the System

In this study, the total number of pollen grains to be located in the 135 samples (video) of the test set was 1234. Discarding all the location proposals generated by the networks that did not exceed a minimum score of 0.85 and after applying the z-stacking fusion algorithm described in Section 3.4, we obtained for our system the overall localization performance parameters shown in Table 5.

Table 5 shows that more than 98% of the grains were correctly located in all network configurations, which seemed to indicate that the problem was robustly approachable with any of the architectures considered. The highest recall rate was reached in RetinaNet Configuration A, which, in addition to considering the grains with a defined edge, incorporated grains with a blurred appearance. However, on the other hand, it had the lowest precision rate and high inference times.

The number of false positives was kept below 1% in both Faster-type networks, being especially low in the case of Network B, where only three grains were estimated in areas where there was no grain defined in the database. Finally, as already mentioned in Section 3.5, depending on the grain area that was present in the view, grain proposals corresponding to true grains could be generated at the edges ($TP_{E}$), but they did not provide relevant information regarding the operation of the final system, because those grains would be analyzed later when moving the view of the microscope. In view of the precision and recall results and considering the shorter inference time required by the Faster R-CNN implementations, the Faster networks seemed good candidates for our system.

Table 6 compares the performance of the different proposals analyzed in Section 2 with the results obtained in our work. As can be observed, the most remarkable result was the high precision rate obtained in our study (99.03%). The recall rate (98.94%) also exceeded that specified in the rest of the referenced studies. However, as already mentioned in Section 2, it was very difficult to carry out a meaningful comparison of the studies due to the great variability of the datasets used by the authors.

Both the work of Díaz-López et al. [9] and Nguyen et al. [13] used the circle Hough transform (CHT) to locate pollen grains at some stage of the detection algorithm. In order to provide an idea of the performance of this technique on our dataset, we processed it with an algorithm that tried to reproduce the location stage used by Nguyen et al. This algorithm, implemented with OpenCV [36], uses a single gray-scaled image per sample, then blurs the image using the median filter, finds circles in the image using OpenCV’s CHT, and finally, applies NMS. The selected image of each sample was the one with the most grains labeled. We studied the filter size and the parameters of the HoughCircles function to maximize the number of correct detections using an IoU > 0.5 as the success criterion.

After several tests, we chose a filter with a size of 17 pixels and the following configuration parameters for the CHT: the same resolution for the accumulator as the input image, a minimum distance between centers of 25 pixels, a gradient of 50, an accumulator threshold for the circle centers of 30, and a radius between 20 and 200 pixels. Under these conditions, we obtained 985 TP, 196 FP, and 249 FN. Hence, the performance of this detection algorithm achieved a recall rate of 79.8% and a precision rate of 83.4%, results quite lower than those obtained by any of our networks. This configuration is reflected in Table 6 as CHT on our dataset.

The errors observed using our CHT-based implementation were due, among others, to: grains that did not have a circular appearance, grains with low contrast to the tinted background, or backgrounds with high noise due to the adhesive surface. These challenges were correctly managed by our system based on deep learning.

### 4.3. Precision in Grain Location and Size Estimation

Table 7 shows the accuracy in the location and size of the predictions made by each system in terms of IoU. The table shows the average values of IoU along with the number of correct proposals generated by the networks for each species and globally. For reference, an IoU of 1.0 accounts for the total accuracy in both the location and size of the object, and as mentioned in Section 3.3, a value of 0.89 is indicative of a high accuracy in both position and size. As Table 7 shows, RetinaNet implementations seemed to provide better adjustment of grain position and dimension, so they would be more suitable in precision applications. Although some of the papers referenced in Section 2 addressed the location and the estimation of the grain size, it was impossible to make a comparison with our results regarding these aspects, due to the absence of any metric in those works.

### 4.4. Graphical Results

Although the numerical results allowed us to get a general idea about the performance of the system, certain details of operation were not appreciated numerically. Therefore, some visual results are shown in this section. As we will see, the images shown in different focal planes allowed us to obtain a spatial idea of the performance of the system and visualize some of the inherent problems of the image space with which we worked.

In the following images, the correct detections are marked by a green BBox, the false positives are specified in red, and finally, the false negatives are marked in blue. The position of the BBox is stored in the database associated with the frame in which it obtained the highest score, so in the focal plane shown, it can be seen as slightly displaced if the associated grain appears visually blurred. In any case, it exceeded the IoU value established in the experiment if it was identified as a TP.

As expressed by the numerical results previously shown, our system was able to efficiently locate almost all of the grains present in the defined test set. Moreover, the yield seemed to show a high independence with respect to the type of grain contained in the sample, the conditions of background complexity, or backlighting contemplated. However, there were certain non-controllable situations in the real samples that caused the system to ignore certain grains, or generate proposals that exceeded the minimum score in areas where no grain actually existed.

**Figure 9:**
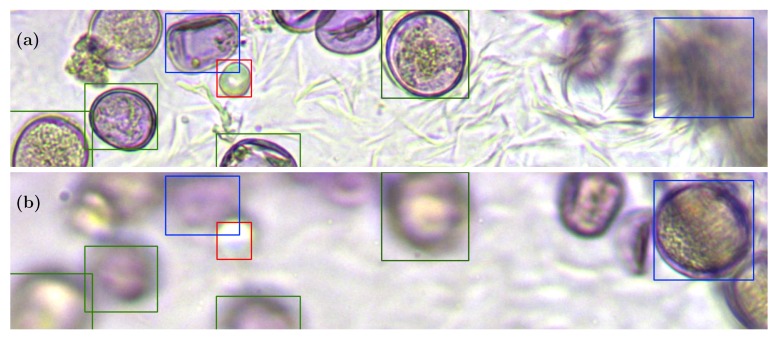
Two cuts in different focal planes that show two different errors: a false positive (red) that has a bubble marked as a pollen grain (**a**) and a false negative (blue box on the right) whose focal plane is far from those studied (**b**).

Thus, Figure 9 obtained using Network B showed two different focal planes of the same sample, in which the system had wrongly identified a bubble as a grain of pollen and two false negatives. One of the false negatives was associated with a grain located on the upper edge of the visible field (next to the bubble), and the other, located in the upper right corner, was highly occluded by the adhesive substrate and was only visible in a frame that was distant from the main focal plane, as shown in Figure 9b.

**Figure 10:**
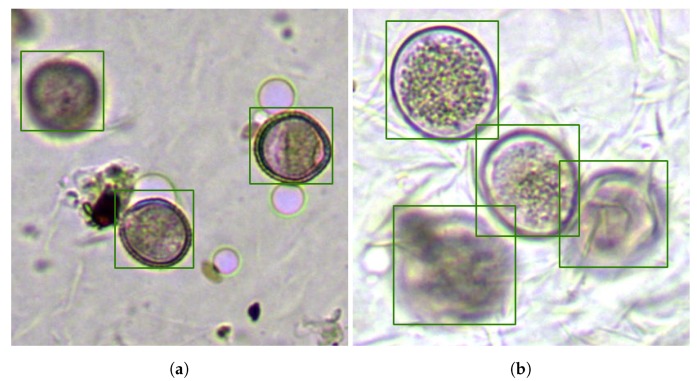
Two examples of grain location on samples of *Olea* (**a**) and *Lolium* (**b**) in which some of the problems identified have been successfully managed: (**a**) ignored debris, bubbles, and dust and (**b**) overlap between grains and highly-different focal planes for each grain.

Despite the result shown in Figure 9, our system seemed to differentiate efficiently the bubble concept from the pollen grain concept; in fact, Network B only generated three false positives. As an example, Figure 10a shows a section of an *Olea* sample with several bubbles, debris, and dust that has been ignored, while the three visible *Olea* grains in the view have been correctly located. Note also the apparent error in the location of the fuzzy grain in the upper left corner, which was due to the choice of the focal plane shown. Additionally, Figure 10b shows a section of a sample of *Lolium* in which the four visible grains have been located correctly even though they presented distant focal planes and two of them appeared grouped, exhibiting deformation of the edge.

**Figure 11:**
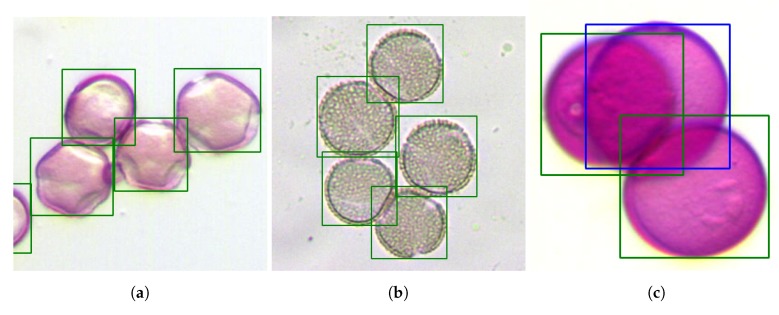
(**a**) A sample of the successful location of all *Quercus* grains despite distinct visible morphology, (**b**) a second sample of *Olea* with different levels of tinting and successful management despite the deformation of the edge, and (**c**) the highly overlapping sample of *Dactylis*m which did not allow the separation of the central grain.

Figure 11a shows another set of grains of the *Quercus* species where, in spite of the variations in visual appearance, the four visible and grouped grains have been located satisfactorily. Figure 11b also shows the correct operation for a cut of an *Olea* sample with different levels of tinting to that shown in Figure 10a and strongly grouped. Finally, Figure 11c shows the result when processing a sample where the great overlap between the two upper grains made the system unable to separate the two overlapping grains.

## 5. Conclusions

The reliable location of pollen grains in tinted adhesive samples is a complex task. In this work, we approached the application of two of the most recent techniques in artificial intelligence (Faster R-CNN with FPN and RetinaNet), obtaining promising results using low-cost equipment, both in the sample acquisition block and in the computational block.

In our view, this work presented some important contributions regarding the location of pollen grains in real microscope images. First of all, we confirmed that it was possible to carry out a successful end-to-end adjustment of an FPN-based Faster R-CNN and a RetinaNet with a relatively small number of palynological samples.

On the other hand, it is important to remark that we developed a system that improved the detection results exposed in the bibliography, both in terms of recall and precision. In addition, by using a standard overlapping criterion (IoU) to determine the existence of a TP, we eliminated the bias that may be introduced by the choice of other non-standard criteria.

This work also provided a basis for comparison when measuring the accuracy of pollen location proposals generated by detection algorithms, using a metric currently accepted in the field of object location (IoU). As can be seen in Table 7, the average value of the accuracy in the estimation of the position in terms of IoU was 0.89 for Faster-based systems and 0.90 for RetinaNet systems. The lowest IoU average value per species did not fall below 0.84, which suggested that all the networks correctly modeled the grain concept, despite the visual differences that existed between the pollen types considered, tinting of the samples, background types, and backlight levels applied to the samples. Therefore, we could estimate a localization performance quite efficiently, without presenting significant variations when breaking down the yield by pollen type.

Regarding the benefit obtained with the inclusion of blurred pollen grain prototypes in the training set, we observed a worse behavior in terms of the generation of erroneous proposals. In addition, the performance obtained by the system that only used sharp prototypes was very high, despite using a smaller number of grain examples to adjust the network. Therefore, although a marked degradation in the overall performance was not observed, the inclusion of blurred grains to adjust the system did not seem a recommendable training strategy to locate pollen grains.

Finally, with regard to our data, we manually collected a color pollen database from 20 pollen slides that contained 11 pollen types. This database allowed performing z-stacked pollen detection studies, similar to ours. We manually labeled each sample to locate each pollen grain with a BBox in the plane of focus where the border was sharpest. As previously mentioned, due to the volumetric characteristics of a pollen grain, the defined BBox may not adequately represent the dimensions of the grain in a focal plane other than that specified in the database record. The database is available upon request for other researchers (see Supplementary Materials).

Although the images were captured at a resolution of 1280 × 1024 pixels, only the versions scaled at 640 × 512 were used as the system input. None of the algorithms required any additional enhancement process on the input images, which, in addition to reducing the computational cost, accounted for the robustness of the algorithm to deal with this type of images. The results obtained seemed to indicate that this level of resolution was sufficient to make an adequate detection of pollen grains, getting a successful separation from the rest of the elements present in the images.

The low detection times, less than 59 ms per image, allowed us to expect an operation close to the real time if an integration was made with an electronically-controlled microscope. The time required by the servomotors to adjust a new view would be used to process in parallel on the GPU. In this situation, it should be studied whether the number of 21 frames used in this study to analyze each sample was suitable or wasteful, in which case, the overall processing speed would increase.

Finally, the high accuracy rate achieved by our system over the test set and the speed of processing led us to think that this technique could be adequate to develop an automated system for the estimation of pollen concentrations.

Anyway, this work opens a number of further research lines. First, the identification of pollen types can be addressed, either by applying this technique in a single step or, where appropriate, using the predictions obtained as input to a new system that allows identifying efficiently the species of each of the localized grains. Besides, it is necessary to include, in the training of future systems, samples obtained in adverse meteorological conditions, with the aim of studying the applicability of this technique to samples with a large amount of dust and debris. As a final objective, it would be useful to integrate a computer-controlled microscope into the system to accelerate and mechanize the sampling process, increasing the reliability of the results obtained and preparing the system for the application to a real environment.

## Figures and Tables

**Table 1:** Overview of recently-published results about automatic pollen grain localization.

	Year	Pollen Types	No. of Slides	No. of Grains	Detector	Recall	Precision
Ranzato et al. [12]	2007	8	N/A	3686	SIFT based	93.9%	8.60%
Landsmeer et al. [10]	2009	N/A	9	65	Color similarity	86%	61%
Nguyen et al. [13]	2013	9	1	768	Active contours	93.8%	89.5%
Redondo et al. [8]	2015	15	N/A	1800	Shape based, human corrected	N/A	N/A
Díaz-López et al. [9]	2015	12	12	4061	Shape and texture	81.92%	18.5%

**Table 2:** Composition of the pollen database showing the registered samples, the number of grains labeled, and complete grains identified by species.

Pollen Types	Samples	Grains Labeled	Full Grains
*Cupressus*	22	91	63
*Avena sativa*	15	86	65
*Avena sterilis*	17	105	84
*Cedrus*	40	598	389
*Dactylis*	60	155	145
*Lolium*	20	261	200
*Olea*	59	505	450
*Phalaris*	20	123	95
*Plantago*	40	344	308
*Platanus*	20	911	753
*Quercus*	73	1056	823
Total	386	4235	3375

**Table 3:** Characteristics of the datasets established to adjust and test the classifier.

Set	Samples	Full Grains	Frames
Train	251	2037	497
Test	135	1234	2863

**Table 4:** Summary of training and inference times for configured networks. Type A networks include blurred grains, while Type B networks only include sharp grains.

Network	Training Time	Inference Time	Mean Inference Time
	(hours)	(s)	(ms)
Faster R-CNN A	3:12	166.7	58
Faster R-CNN B	3:11	169.8	59
RetinaNet A	7:19	280.3	98
RetinaNet B	7:17	293.7	103

**Table 5:** Summarized localization performance results (IoU > 0.5, score > 0.85). Percentages are expressed on the actual number of grains to be located (excluding grains partially visible at the edges).

Parameter	Faster A		Faster B		RetinaNet A		RetinaNet B	
	Value	%	Value	%	Value	%	Value	%
Predictions	1277	n/a	1252	n/a	1295	n/a	1257	n/a
TP	1221	98.94	1216	98.54	1226	99.35	1,210	98.06
FN	13	1.05	18	1.46	8	0.65	24	1.94
FP	12	0.97	3	0.24	21	1.70	12	0.97
$TP_{E}$	44	3.56	33	2.67	48	3.89	35	2.83
Recall	-	98.94	-	98.54	-	99.35	-	98.06
Precision	-	99.03	-	99.75	-	98.31	-	99.02

**Table 6:** Comparison with the latest works published about automatic pollen grain localization. An N/A in a field indicates the non-existence of the data in the work or the impossibility of knowing if any sample has been used in the task. CHT, circle Hough transform.

	Pollen Types	No. of Slides	No. of Grains Train	No. of Grains Test	Detector	Recall	Precision
Ranzato et al. [12]	8	N/A	0	3686	SIFT based	93.9%	8.60%
Landsmeer et al. [10]	N/A	9	N/A	65	Color similarity	86%	61%
Nguyen et al. [13]	9	1	N/A	768	Active contours	93.8%	89.5%
Díaz-López et al. [9]	12	12	N/A	4,061	Shape and texture	81.92%	18.5%
CHT on our dataset	11	20	2037	1234	CHT	79.82%	83.40%
Our method	11	20	2037	1234	F.R-CNN, z-stacks	98.94%	99.03%

**Table 7:** Accuracy in the location of pollen grains for the classes studied, expressed in terms of IoU between the predictions with the ground-truth bounding boxes of real grains. The standard deviation is indicated in parentheses.

Pollen Types	Faster A		Faster B		RetinaNet A		RetinaNet B	
	IoU (σ)	Pred.	IoU (σ)	Pred.	IoU (σ)	Pred.	IoU (σ)	Pred.
*Cupressus*	0.90 (0.03)	29	0.90 (0.04)	29	0.93 (0.02)	29	0.90 (0.03)	29
*Avena sativa*	0.86 (0.09)	17	0.88 (0.06)	16	0.88 (0.06)	18	0.89 (0.06)	17
*Avena sterilis*	0.86 (0.07)	43	0.88 (0.05)	43	0.89 (0.06)	43	0.90 (0.06)	43
*Cedrus*	0.91 (0.03)	162	0.91 (0.02)	162	0.93 (0.02)	162	0.93 (0.02)	162
*Dactylis*	0.89 (0.06)	59	0.91 (0.05)	59	0.91 (0.06)	60	0.92 (0.05)	60
*Lolium*	0.89 (0.04)	82	0.88 (0.03)	80	0.91 (0.04)	82	0.91 (0.03)	75
*Olea*	0.89 (0.04)	194	0.89 (0.04)	192	0.90 (0.04)	194	0.90 (0.04)	191
*Phalaris*	0.94 (0.03)	36	0.93 (0.02)	36	0.93 (0.04)	36	0.94 (0.01)	36
*Plantago*	0.89 (0.04)	116	0.89 (0.04)	116	0.89 (0.04)	116	0.89 (0.03)	116
*Platanus*	0.85 (0.04)	180	0.86 (0.03)	180	0.84 (0.04)	180	0.86 (0.03)	180
*Quercus*	0.90 (0.03)	303	0.91 (0.02)	303	0.90 (0.03)	306	0.91 (0.03)	301
Mean per grain	0.89 (0.04)	1221	0.89 (0.04)	1216	0.90 (0.05)	1226	0.90 (0.04)	1210

## Supplementary Materials

The following are available online at http://capi.unex.es/pollendb1, Dataset D1: CAPIPollen DB1.
